# Millipore xMap® Luminex (HATMAG-68K): An Accurate and Cost-Effective Method for Evaluating Alzheimer's Biomarkers in Cerebrospinal Fluid

**DOI:** 10.3389/fpsyt.2021.716686

**Published:** 2021-08-31

**Authors:** Erika Oliveira Hansen, Natalia Silva Dias, Ivonne Carolina Bolaños Burgos, Monica Vieira Costa, Andréa Teixeira Carvalho, Antonio Lucio Teixeira, Izabela Guimarães Barbosa, Lorena Aline Valu Santos, Daniela Valadão Freitas Rosa, Aloisio Joaquim Freitas Ribeiro, Bernardo Mattos Viana, Maria Aparecida Camargos Bicalho

**Affiliations:** ^1^Jenny de Andrade Faria Institute– Reference Center for the Elderly, Hospital das Clínicas, Universidade Federal de Minas Gerais, Belo Horizonte, Brazil; ^2^Molecular Medicine Program, Faculdade de Medicina, Universidade Federal de Minas Gerais, Belo Horizonte, Brazil; ^3^Neuroscience Program, Institute of Biological Sciences, Universidade Federal de Minas Gerais, Belo Horizonte, Brazil; ^4^Elderly Psychiatry and Psychology Extension Program (PROEPSI), Faculdade de Medicina, Universidade Federal de Minas Gerais, Belo Horizonte, Brazil; ^5^Adult Health Sciences Applied Program, Faculdade de Medicina, Universidade Federal de Minas Gerais, Belo Horizonte, Brazil; ^6^René Rachou Institute, Oswaldo Cruz Foundation (Fiocruz), Belo Horizonte, Brazil; ^7^Department of Psychiatry and Behavioral Sciences, UT Health, Houston, TX, United States; ^8^Instituto de Ensino e Pesquisa, Santa Casa de Belo Horizonte, Belo Horizonte, Brazil; ^9^Department of Mental Health, Faculdade de Medicina, Universidade Federal de Minas Gerais, Belo Horizonte, Brazil; ^10^National Institute of Science and Technology of Molecular Medicine (INCT-MM), Faculdade de Medicina, Universidade Federal de Minas Gerais, Belo Horizonte, Brazil; ^11^Institute of Exact Sciences, Statistics Department, Universidade Federal de Minas Gerais, Belo Horizonte, Brazil; ^12^Department of Clinical Medicine, Faculdade de Medicina, Universidade Federal de Minas Gerais, Belo Horizonte, Brazil

**Keywords:** Alzheimer's disease, biomarker, Millipore xMap^®^ Luminex, cerebrospinal fluid, Aβ42, tau, INNOTEST

## Abstract

**Background:** Alzheimer's disease (AD) biomarkers are of great relevance in clinical research, especially after the AT(N) framework. They enable early diagnosis, disease staging and research with new promising drugs, monitoring therapeutic response. However, the high cost and low availability of the most well-known methods limits their use in low and medium-income countries. In this context, Millipore xMap^®^ Luminex may be a cost-effective alternative. In our study, using INNOTEST^®^ as reference, we assess the diagnostic accuracy of Millipore xMap^®^ and propose a cutoff point for AD.

**Methods:** We performed lumbar puncture of seven older individuals with clinically defined AD, 17 with amnestic mild cognitive impairment (aMCI) and 11 without objective cognitive impairment-control group (CG). Cerebrospinal fluid (CSF) biomarkers concentrations for aB42, p-Tau, and t-Tau were measured by INNOTEST^®^ and Millipore xMap^®^, and then the techniques were compared to assess the diagnostic accuracy of the new test and to define a cutoff.

**Results:** INNOTEST^®^ and Millipore xMap^®^ measurements showed all correlations >0.8 for the same biomarker, except for t-Tau that was 0.66. Millipore xMap^®^ measurements showed a robust accuracy for all biomarkers, with AUC higher than 0.808 (t-Tau), and the best for Aβ42 (AUC = 0.952). The most accurate cutoffs were found at 1012.98 pg/ml (Aβ42), 64.54 pg/ml (p-tau), 3251.81 pg/ml (t-tau), 3.370 (t-Tau/Aβ42), and 0.059 (p-Tau/Aβ42).

**Conclusion:** Given its good accuracy and cost-effectiveness, Milliplex xMap^®^ tests seems a reliable and promising tool, especially for low and middle-income countries.

## Introduction

Globally, over 50 million people were living with dementia in 2018, and this number is expected to nearly triple to 152 million by 2050 (1). This growth stands out in low and middle-income countries (2). The global cost of dementia was about 818 billion in 2015, with only about 10% incurred in low and middle-income countries (3). Following this trend, in the Southern Latin American, it is estimated a 77% increase in the number of people with dementia. As prevalence rates across the region increase, so too will the costs associated with providing dementia care and support (4). Dementia is recognized as one of the main causes of functional decline, morbidity and mortality among elderly (5).

Alzheimer's disease (AD) is the most common cause of dementia (2). It is associated with the accumulation of insoluble forms of amyloid-β (Aβ) and aggregation of tau protein in neurofibrillary tangles (6). The clinical diagnosis in early stages is often delayed since it may rely on patients' signs and symptoms or caregivers' concerns, as well as cognitive assessment. In research centers, a clinical diagnosis of AD is around 80% sensitive and 70% specific based on clinicopathological studies (7). Therefore, the use of biomarkers is of great relevance, making it possible to establish early diagnosis, estimating risks, assessing disease stages and monitoring progression and therapeutic response.

In 2018, the National Institute on Aging and Alzheimer's Association proposed a research framework focused on the diagnosis of AD with biomarkers (8). It is a binary system in which biomarkers are grouped into those related to β amyloid deposition (A), tau pathology (T), and neurodegeneration (N) - [AT(N)], based on cerebrospinal fluid (CSF) and/or molecular and structural neuroimaging (9). Therefore, diagnosis is not only based on clinical manifestations of AD, so that the definition of AD has become a biological construct. Based on this background, the AT(N) framework defined three categories: Normal AD biomarkers, Alzheimer's continuum and Non-AD pathologic change (8).

The term Alzheimer's continuum was first established in 2011 by Sperling et al. (10). Nowadays, it is well-established that AD is a continuum from a preclinical to a symptomatic stage, and that neuropathological changes precede clinical manifestations by 20–30 years. Updating concepts, Alzheimer's continuum is an umbrella term that include all individual with biomarkers evidence of Aβ deposition, independently from tau pathology or neurodegeneration, and the term “Alzheimer's disease” should be used only if there is evidence of both Aβ and tau pathology. This concept is independent of the clinical presentation (8).

Nowadays, there are available commercial techniques for CSF biomarkers, like the non-automated method enzyme-linked immunosorbent assay (ELISA), the semi-automated Luminex xMAP (11) and the next generation automated assays, chemiluminescent enzyme immunoassay (Lumipulse^®^, Fujirebio, Europe) and electrochemiluminescence immunoassay (Elecsys^®^, Roche Diagnostics, Switzerland) (12, 13). Despite the emergence of innovative technology, the most commonly used technique in research has been ELISA, particularly INNOTEST^®^ (Fujirebio, Europe) (14). This method is based on solid-phase enzyme immunoassay by a single analyte (15). On the other hand, Luminex xMap is a multiple analyte test which simultaneously detects and quantifies both Aβ and Tau proteins in the same sample (16). Therefore, this method has advantages in reducing sample volume, processing time and decreases chance of human errors (17). While additional advantages of xMap might include reduced intra-assay and intra-laboratory variations, the method displays worse inter-assay performance than automated testing (18). Taking into account cost-effectiveness and accuracy, the Luminex xMAP can be an interesting alternative.

Previous research showed similar accuracy between Luminex xMAP and ELISA (19–22). Until now, the most commonly used Luminex xMap in research has been INNO-BIA AlzBio3^®^ (Fujirebio, Europe). However, the company Fujirebio discontinued its commercialization in January 2021. So, other commercially available Luminex tests should be used, like Millipore xMap^®^ (HNABTMAG-68K) (Millipore, Germany). To the best of our knowledge, Millipore xMap^®^ was little studied as an Alzheimer's biomarker and there is no validation of this method in AD.

The aim of this study was to assess the correlation and accuracy of Millipore xMap^®^ for AD's CSF biomarker, using ELISA INNOTEST^®^ as gold-standard. Furthermore, we propose Millipore xMap^®^ cutoff points for AD's diagnosis in clinical scenarios based on ROC curves analysis.

## Materials and Methods

We recruited older adults who attended the geriatric outpatient clinic from the Jenny de Andrade Faria Institute (JAFI) - Hospital das Clínicas - Universidade Federal de Minas Gerais (HC-UFMG), which including 24 patients with cognitive decline, amnestic mild cognitive impairment (aMCI) and probable Alzheimer's disease dementia (ADD), and two participants without objective cognitive impairment. IJAF is a Reference Center for the diagnosis and treatment of dementia and for diagnosis and treatment of frailty older adults. In addition, we included nine patients without cognitive impairment who were recruited from scheduled orthopedic surgery.

All subjects underwent a comprehensive clinical, cognitive and behavioral assessment (23) by a previously trained geriatrician or geriatric psychiatrist. Briefly, the screening tests included Mini-mental state examination (24), Dementia Rating Scale (25), Clinical Dementia Rating Scale (26), Neuropsychiatric Inventory (27) and Instrumental Activities of Daily Living (28). Individuals with cognitive impairment also underwent a neuropsychological assessment and have been followed at the same Center for two or more years. The neuropsychological battery used was previously validated for assessment of older adults with low educational level and with heterogeneous cognitive background (29). All tests were validated for use with Brazilian elderly and cut-off points were considered according to their education level. Patients with cognitive impairment also underwent routine blood tests (e.g., hematology, biochemistry, thyroid-stimulating hormone, vitamin B12 and folate levels, syphilis, and HIV serology) and brain imaging studies (computerized tomography or nuclear magnetic resonance).

Diagnosis of probable ADD was performed according to the criteria of McKhann et al. (30) and of aMCI according to the DSM-5 (31). So, we defined three groups according to the clinical diagnosis: 11 individuals without objective cognitive impairment-control group (CG), seven with probable mild ADD and, 17 with aMCI.

The research was approved by the local Ethics Committee (CAAE 79354317.1.0000.5149) and all participants or legal guardians signed the consent form.

### CSF Sample Collection

Lumbar puncture (LP) was performed by an anesthesiologist to collect cerebrospinal fluid (CSF) from participants with cognitive impairment. For CG, CSF collection was performed during lumbar puncture for spinal anesthesia, since these patients would be submitted to elective surgical procedures.

LP was performed into the L3/L4 or L4/L5 intervertebral space to collect 6 ml of CSF in polypropylene tubes. Samples were immediately transported to the laboratory, where they were centrifuged at 3,000 revolutions per minute (rpm) for 15 min, at 4°C, at a maximum time of 2 h after collection. Then, they were frozen and stored at −80°C until the time of analysis.

Concentrations of Aβ1-42, t-tau and p-tau in CSF samples were measured using both ELISA and Luminex xMap assay protocols. Samples were processed by trained professionals, based on robust protocol, as, our laboratory has a strict quality control system. Also, we followed the protocols described by manufactures We used the INNOTEST hTAU Ag^®^, INNOTEST PHOSPHO-TAU (181P)^®^, and INNOTEST β-Amyloid (1-42)^®^ (Fujirebio, Europe) kits by ELISA technique. We performed the Human Amyloid Beta and Tau Magnetic Bead Panel^®^ (HNABTMAG-68K) (Millipore, Germany) kit by Luminex xMAP technique. This technology uses labeled microspheres or beads and internally dyes bead sets with precise concentrations of fluorescent dyes, resulting in 500 distinctly colored bead sets. The bead mixture is incubated with the sample and the median fluorescence intensities detected on a Luminex instrument (32).

The enzymatic immunoassay ELISA - INNOTEST^®^ was used as a reference test for biological diagnosis. This is a solid-phase enzyme immunoassay in which the protein is captured by a monoclonal antibody. CSF samples are added and incubated with a biotinylated antibody, and then this antigen-antibody complex is detected. After addition of substrate working solution, samples develop a specific color. The color intensity is a measure for the amount of protein in the sample (33).

The cut-off points for ELISA – INNOTEST^®^ was based on Duits et al. work (34, 35): Aβ42 <550 pg/ml, total tau (t-tau) > 375 pg/ml, phosphorylated tau (p-tau) > 52 pg/ml, ratio t-tau/Aβ42 > 0.52 and ratio p-tau/ Aβ42 > 0.08. Thus, we categorize patients into biological groups according to the AT(N) system (10): (1) normal AD biomarkers (A-T-N-), (2) Alzheimer's continuum (A+T(+/–)N(+/–) or (3) Non-AD pathologic change (A-T+N(+/–) or A-T-N+). Lastly, we compare each biomarker's results of ELISA and Luminex to assess the accuracy and define a possible cut-off for Millipore xMap^®^.

### Statistical Analysis

Socio-demographic, comorbidities and biomarkers differences between clinical and biological groups were assessed using chi-square tests (with *p*-values chosen by MonteCarlo simulation), ANOVA and the Kruskall-Wallis tests for non-parametric variables. For multiple comparisons, we applied the Turkey method or the Nemenyi test with X square approximation. We analyzed compared variables based on both clinical and biological criteria. CSF biomarkers were log transformed. Pearson's linear correlation showed the strength of association between the same biomarker in the two methods.

Receiver Operating Characteristic (ROC) curves were plotted and areas under the curve (AUCs) were calculated for every biomarker and index, and the cut-off points of Luminex were established for the best combination of sensitivity and specificity.

We also applied the following precision measures: proportion of correct classifications, Youden index and the distance from the cutoff point to the top left of the ROC curve.

Finally, we calculate the power of the test using the power.roc.test function from the pROC package of the R software (36).

Statistical analyzes were performed using the R software (www.r-project.org).

## Results

We studied 35 elderly individuals, 11 without objective cognitive impairment, seven with probable ADD and 17 aMCI. The mean age was 72.63 years (SD 6.02), 82.85% were female, 5.27 years (3.38) of formal education and 42.85% had family history of dementia. The results were described considering clinical and biological criteria (based on results of Aβ42, p-tau and t-tau ELISA). Results of clinical and socio demographic variables are shown in Table 1. There were no statistically significant differences among groups regarding age, gender, years of formal education, family history of ADD. As expected, ADD had significantly lower MMSE and higher Pfeffer scores. Also, ADD, and aMCI subjects had significantly more depressive symptoms. These differences were not present between the groups by biological criteria. There was 85.7% of agreement between the clinical and biological diagnosis of ADD.

**Table 1:** Clinical and socio demographic characteristics according to baseline diagnosis clinical and biological.

**Variables**	**Clinical diagnosis**				**Biological diagnosis**			
	**CG** **(11)**	**ADD** **(7)**	**aMCI** **(17)**	***p*-value**	**Normal AD** **biomarkers** **(10)**	**Alzheimer's** **continuum** **(14)**	**Non-AD** **pathologic change** **(11)**	***p*-value**
Age (years)	70.82 (6.11)	77.0 (5.94)	72.0 (5.41)	0.084	71.10 (5.38)	74.14 (5.86)	72.09 (6.82)	0.458
Formal education (years)	6.18 (4.07)	4.14 (2.04)	5.15 (3.34)	0.594*	4.60 (1.35)	4.96 (4.35)	6.27 (3.29)	0.286*
Gender** (%) M/F	9.1/90.9	42.86/57.14	11.77/88.23	0.1784	20.0/80.0	21.43/78.57	9.1/90.90	0.7226
MMSE	26.18 (2.48)	19.29 (3.82)	22.76 (3.54)	0.002*	24.10 (3.11)	21.71 (4.55)	24.09 (3.94)	0.240*
Peffer	0.44 (0.73)	8.71 (4.82)	3.23 (2.59)	0.000*	2.43 (2.07)	4.62 (5.28)	3.33 (3.77)	0.690*
Parental ADD (%)**	36.36	57.14	41.12	0.7626	40.00	50.00	36.36	0.8351
Smoking (%)**	81.82	57.14	47.06	0.2149	60.00	57.14	63.64	0.1082
BMI	28.88 (4.81)	25.04 (4.40)	28.37(5.12)	0.240	30.42 (5.46)	26.70 (4.24)	27.03 (4.95)	0.156
Stroke (%)**	18.18	14.28	5.89	0.8081	20.00	14.28	0.00	0.4398
CAD (%)*	18.18	0.00	17.64	0.6822	20.00	14.28	9.09	0.5090
Diabetes mellitus (%)**	45.45	0.00	52.94	0.0615	50.00	28.57	45.45	0.5827
Dyslipidemia (%)**	63.63	42.85	52.94	0.7386	60.00	42.85	63.63	0.6192
Depressive symptoms (%)	18.18	71.42	70.59	**0.0159**	60.00	57.14	54.54	0.8256

*Kruskal-Walis test.

**X-square test.

*CG, individuals without objective cognitive impairment (control group); ADD, Alzheimer's disease dementia; aMCI, amnestic mild cognitive impairment; M, male, F, female; MMSE, Mini Mental State; BMI, body mass index; CAD, coronary arterial disease. Bold value means p values < 0.05*.

Results of CSF biomarkers are shown in Table 2. We observed that the p-Tau/Aβ42 ratio and t-Tau/Aβ42 ratio had significant differences between clinical criteria groups in both tests, although, there were no statistically significant differences for Aβ42 alone. This difference was observed between CG and ADD groups for both ratios, and aMCI and ADD for p-Tau/Aβ42. For biological groups, we found significant differences in all biomarkers for both techniques (except for Aβ42 by ELISA and Luminex and t-Tau by Luminex), particularly for ratio.

**Table 2:** Concentrations of biomarkers (Aβ42, p-Tau, and t-Tau) by ELISA and xMAP in cerebrospinal fluid according to baseline diagnosis clinical and biological.

**Variables**	**Clinical diagnosis**				**Biological diagnosis**			
	**CG** **(11)**	**ADD** **(7)**	**aMCI** **(17)**	***p*-value**	**Normal AD** **biomarkers** **(10)**	**Alzheimer's** **continuum** **(14)**	**Non-AD** **pathologic change** **(11)**	***p*-value**
Aβ42 ELISA	759.09 (280.93)	517.31 (54.49)	740.79 (314.64)	0.111	947.89 (245.20)	426.10 (85.26)	829.12 (240.47)	**0.000**
Aβ42 xMAp	1483.32 (692.25)	993.97 (756.84)	1195.14 (575.70)	0.148	1506.10 (584.88)	739.14 (180.66)	1652.98 (710.06)	**0.000**
p-TAU ELISA	57.29 (24.06)	88.28 (32.27)	51.90 (26.05)	**0.018**	38.64 (6.73)	67.51 (35.74)	72.92 (23.39)	**0.007**
p-TAU xMap	71.48 (32.33)	131.91 (46.07)	83.48 (52.60)	0.023*	58.09 (14.06)	99.54 (56.75)	104.93 (51.67)	0.043*
t-TAU ELISA	312.43 (129.43)	578.93 (227.31)	353.43 (212.64)	**0.025**	251.30 (67.16)	422.63 (249.17)	460.70 (207.33)	**0.038**
t-TAU xMap	4028.15 (2817.22)	5125.86 (2072.83)	4231.68 (2737.00)	0.410	2790.35 (792.89)	4269.01 (2680.45)	5859.96 (2708.46)	**0.021**
p-TAU/Aβ42 ELISA	0.09 (0.05)^1^	0.20 (0.10)^1;5^	0.08 (0.05)^5^	**0.004**	0.04 (0.01)^a^	0.16 (0.09)^a^	0.10 (0.05)	**0.000**
p-TAU/Aβ 42 xMap	0.06 (0.06)^2^	0.18 (0.12)^2;6^	0.08 (0.07)^6^	**0.017**	0.04 (0.02)	0.14 (0.10)	0.09 (0.08)	**0.019**
t-TAU/Aβ42 ELISA	0.47 (0.28)^3^	1.37 (0.77)^3;7^	0.54 (0.39)^7^	**0.003**	0.27 (0.08)	1.02 (0.66)	0.63 (0.43)	**0.000**
t-TAU/Aβ42 xMap	3.27 (3.19)^4^	6.80 (3.81)^4^	3.94 (2.65)	**0.041**	2.01 (0.76)	5.97 (3.59)	4.27 (3.06)	**0.002**

*Kruskal-Walis test. Multiple comparison: ^1^p = 0.014; ^2^p = 0.0158; ^3^p = 0.0039; ^4^p = 0.0315; ^5^p = 0.0037; ^6^p = 0.0415; ^7^0.056. ^a^p = 0.000. CG, individuals without objective cognitive impairment-control group; ADD, Alzheimer's disease dementia; aMCI, amnestic mild cognitive impairment. Bold value means p values < 0.05.

**Figure 1:**
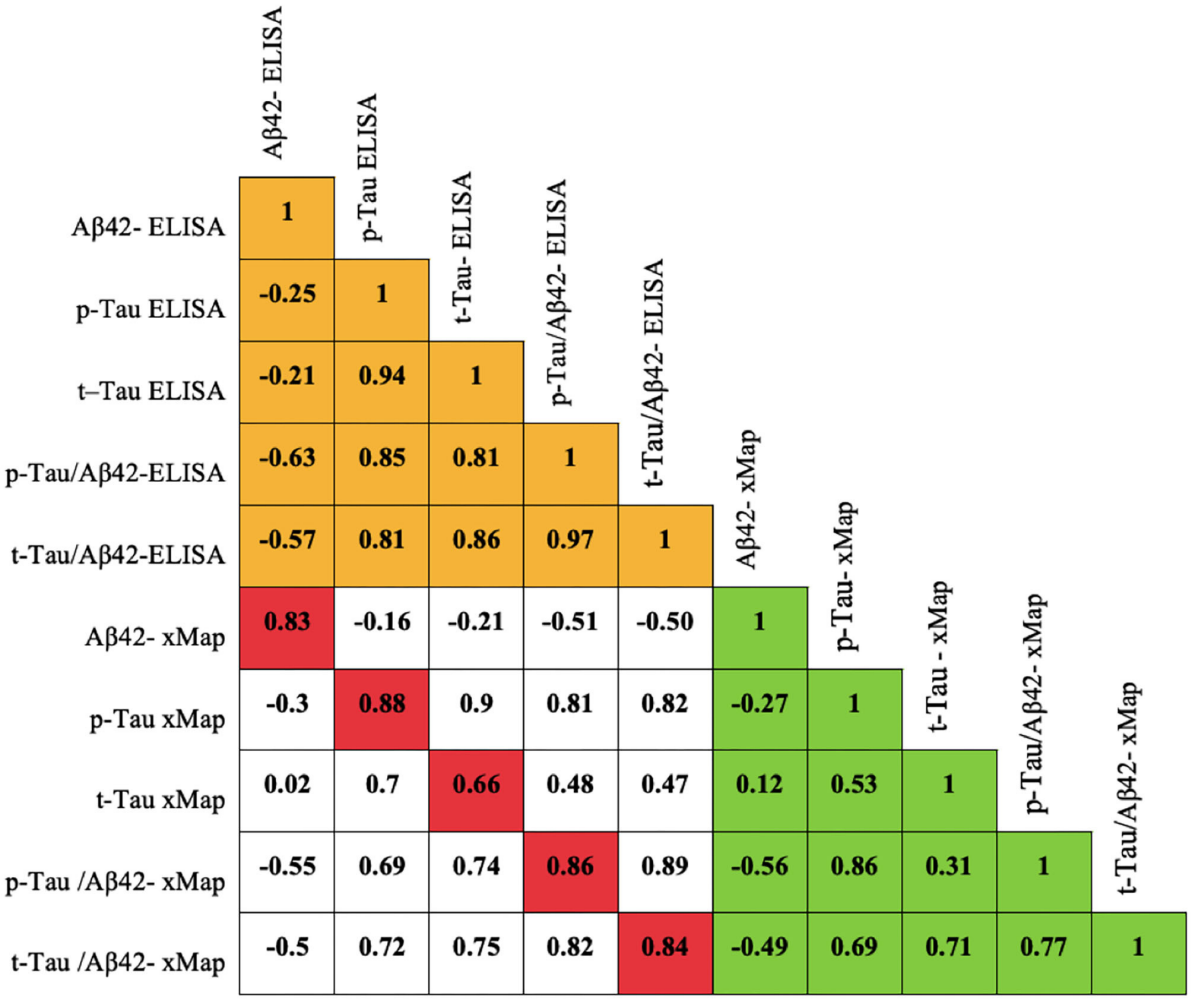
Pearson correlation matrix for biomarkers measured by the ELISA and Millipore xMap^®^ methods.

INNOTEST^®^ and Millipore xMap^®^ measurements showed strong correlations for the same biomarker. All correlations were >0.8, except for t-Tau, indicating a strong linear association between the results of each biomarker (Figure 1).

**Figure 2:**
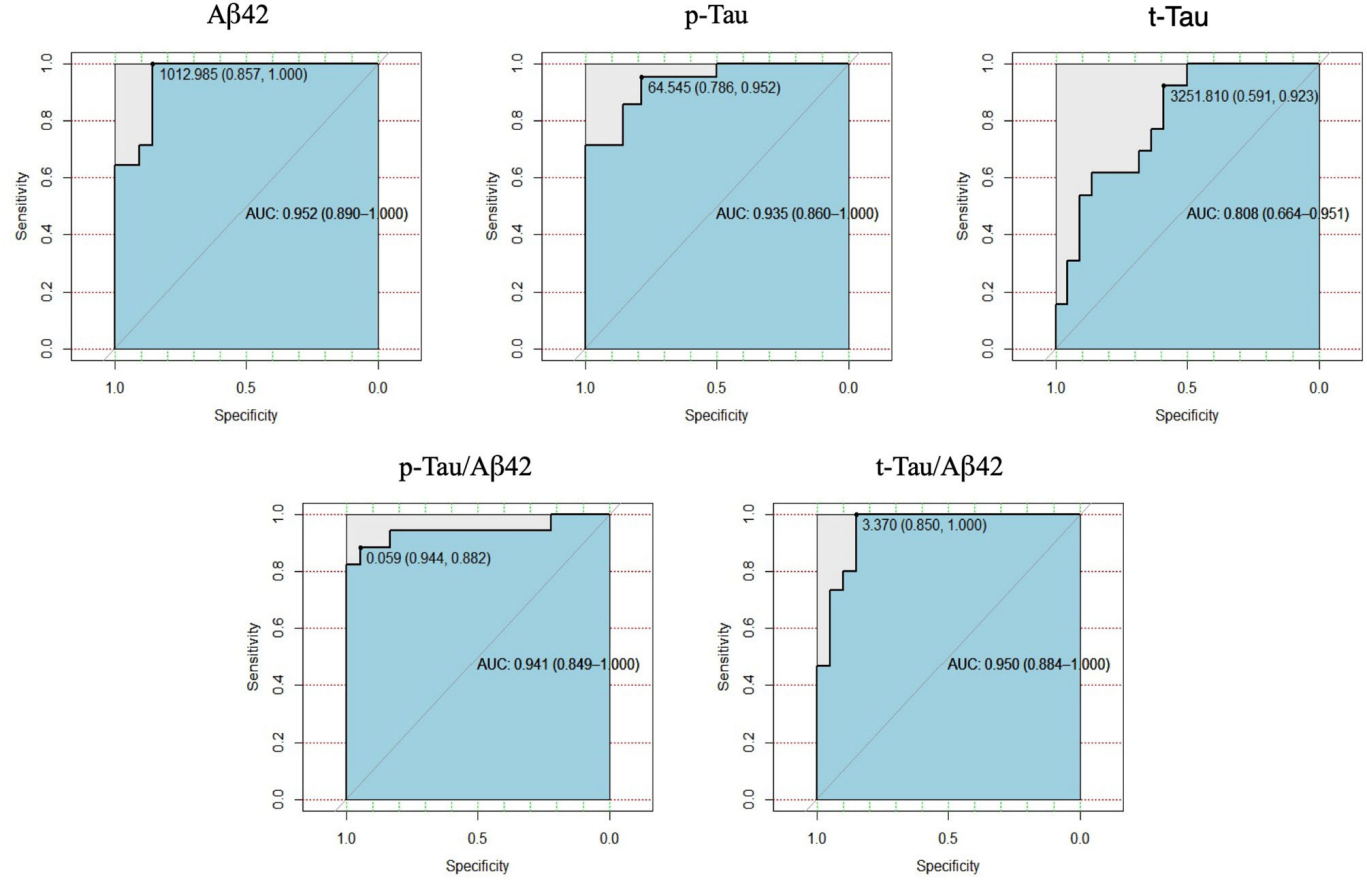
Receiver Operating Characteristics (Roc) curves of Aβ42, p-Tau, t-Tau, p-Tau/Aβ42 e t-Tau/Aβ42 of Millipore xMap^®^, compared with INNOTEST^®^.

The ROC curve analyses using xMap^®^ showed a good diagnostic accuracy for all biomarkers (Figure 2). The highest area under the curve was 0.952 for Aβ42 (sensitivity = 100% and specificity = 85.7%) at the optimal cutoff of 1012.98 pg/ml (Figure 2). This cutoff was determined by the best Youden Index and the distance to the top-left corner of the ROC curve, and so, it maximized the proportion of correct classifications (Table 3). It is important to notice that only t-Tau cutoff did not meet both criteria. In this case, we considered the cutoff 3251.81 pg/ml, based in Youden index, once it had the best sensitivity (Table 3, Figure 2).

**Table 3:** Optimal cut-off of Aβ42, p-Tau, t-Tau, p-Tau/Aβ42, and t-Tau/Aβ42 by Millipore xMap®, with their respective values of sensitivity, specificity, precision measurements and area below the ROC curve.

**Biomarker**	**Cut-off**	**Sensitivity**	**Specificity**	**Proportion of** **correct** **classifications**	**Youden** **index**	**Distance to** **the top-left** **corner**	**AUC**
Aβ42	1012.98	1.000	0.857	0.9143	1.8571	0.0204	0.952
p-Tau	64.540	0.952	0.786	0.8857	1.7381	0.0482	0.935
t-Tau	3251.810	0.923	0.591	0.7143	1.5140	0.1743	0.808
p-Tau/Aβ42	0.0595	0.882	0.944	0.9143	1.8268	0.0169	0.941
t-Tau/Aβ42	3.370	1.000	0.850	0.9143	1.8500	0.0225	0.950

The lowest AUC value (area under the ROC curve) obtained for the biomarkers considered was 0.80 (for t-Tau). With the sample sizes considered in the study, and considering a significance level of 5%, the power of the test, was at least 0.90.

## Discussion

In this study, we observed that the Millipore xMap^®^ is an accurate diagnostic test, and it shows high concordance with results of INNOTEST^®^. Furthermore, it can rightly discriminate between ADD and elderly without objective cognitive impairment, although, it cannot differentiate from patients with aMCI.

Nowadays, the use of biomarkers has been encouraged as they are likely to play a role in the early diagnosis of AD, differential diagnosis of dementias, treatment and monitoring of new disease-modifying drug (37). AD biomarkers are mainly measured in the CSF and through molecular neuroimaging with PET-CT. Several studies that compared CSF AD biomarkers showed high concordance between amyloid PET (38) with both ELISA and Luminex (39, 40). Some of them indicated CSF biomarkers can detect cerebral amyloid-β accumulation earlier than PET (41). Similar findings have been reported for Tau biomarkers, with moderate association between CSF and PET biomarkers (42). Finally, it is important to notice that lumbar puncture is a well-know, safe, accessible and easy procedure, while PET-CT is an expensive technology, rarely available, which depends on specific substrates, in addition to skilled professionals for manipulate the equipment and interpretation of the results. Thus, in most low and middle-income countries, CSF biomarkers are more cost-effective procedures (43).

ELISA and Luminex are the most used CSF biomarkers techniques, but these techniques generate different absolute measures for CSF Aβ 42 (38). Several reports showed good correlation between them (19–22). Applying the Milliplex xMap^®^, we observed higher levels of all biomarkers than INNOBIA ALZBIO3^®^ and INNOTEST^®^. However, measurements of each biomarkers followed the expected trend: reduction of Aβ42 and increase of t-Tau and p-Tau in patients with ADD compared to cognitively intact controls. Also, the strong correlation found between both methods reinforces the consistency of our results with previous clinical studies.

It is important to notice that biomarker levels and cutoff values are still a matter of debate in the literature. More than 10% of individuals without cognitive impairment may have positive CSF AD biomarkers, especially after 60 years-old (44). In a large cohort of healthy control subjects study, 42% of subjects over 50-years-old had abnormal CSF Aβ42, particularly for APOE4 carriers, and t-Tau and p-Tau increased from the sixth decade of life independent of APOE4. It is expected that frequency of both positive biomarkers (amyloid and neurodegeneration) increases to 28% at 85 years (45). In this context, some authors suggest there should be an age-adjusted cut-off for t-tau biomarker (46). On the other hand, Toledo et al. (45) argued these changes most likely represent an increase in frequency of preclinical AD, and therefore, they considered the cut-off should not be adjusted based on age. Lastly, members of the Alzheimer's Biomarkers Standardization Initiative (ABSI) suggested the use of a “gray zone” defined as a 10% increment of the cutoff value in the case of t-Tau and p-Tau or a 10% decrement of Aβ42 (47).

Another way to improve accuracy of biomarkers is through the use of ratios. Several studies have attempted to establish mathematical formulas to improve diagnostic accuracy. Duits et al. (34) suggested that using the ratio t-Tau/Aβ 42 for ELISA assays performed as good as complex regression formulas, and it recommended applying this rate for differentiating AD patients from other dementia (34). Furthermore, it has been suggested that the ratio could be used not only for differential diagnosis of dementia but also for identifying MCI that will convert to ADD (48, 49). In our study we found p-Tau/Aβ ratio was able to differentiate between patients with ADD and cognitively intact controls, and ADD and aMCI, for both technologies.

Finally, plasma AD biomarkers represent a more convenient and less invasive alternative to CSF biomarkers. Previous studies showedplasma p-tau181 (50, 51), p-tau217 (52, 53), and p-tau231 (54) isoforms can correctly diagnose and predict AD in large studies. However, this is currently a very expensive technology and, unfortunately, it is not available in low and middle-income countries, where million people are living with dementia.”

We found an association between depressive and cognitive symptoms in the clinical diagnostic group. However, we did not observe it in the biological criteria. Neuropsychiatric symptoms are very common in dementia syndromes and often precede cognitive symptoms (55). A meta-analysis estimated depression affects almost 30% of persons with MCI, with higher prevalence in memory clinics (56). Some studies suggested depressive symptoms in late life increases the risk for dementia and may even be a prodromal feature of ADD (57). Furthermore, some studies demonstrated a link between depressive symptoms and Aβ deposition, suggesting that higher neuropsychiatric symptoms are associated with higher Aβ and cognitive decline in individuals with cognitive impairment over time (58). However, this evidence was not consistent across studies (59).

The main limitations of this study were a relatively small sample. However, sample size proved to be adequate to test the hypothesis under study. Besides, It is not very different from other studies (16). Furthermore, as the definite diagnosis of AD can only be made at autopsy, there is no true gold standard test for determining whether Aβ burden is normal or abnormal *in vivo* (60). Of the current available methods, we considered ELISA as gold standard because it is the most used test in research and clinical settings, and it is highly correlated with PET and histopathologic results (14, 38).

It is important to notice that dementia incidence and prevalence are expected to increase in low and middle-income countries, especially in Latin America. Therefore, there is a need for affordable and cost-effective biomarkers tests for AD in these countries. Luminex kits are approximately half of the cost of ELISA kits, and has a semi-automated and multiple analyte processing, analyzing both Aβ and Tau proteins in the same sample. Therefore, this may represent a scalability advantage of Luminex in a more cost-effective manner.

In conclusion, the use of Milliplex Map as well as other Luminex alternatives may be a suitable technology with good accuracy and cost-effectiveness for clinical and research scenarios, especially for low and middle-income countries. To the best of our knowledge, this is the first study to explore the use of this assay in older adults and to compare it with the current most used technique. Nevertheless, it is important to notice that more research is needed to better understand the limitations and advantages of the Milliplex Map^®^ in different clinical and research scenarios.

## Data Availability Statement

The raw data supporting the conclusions of this article will be made available by the authors, without undue reservation.

## Ethics Statement

The studies involving human participants were reviewed and approved by Faculdade de Medicina da Universidade Federal de Minas Gerais - CAAE 79354317.1.0000.5149. The patients/participants provided their written informed consent to participate in this study.

## Author Contributions

EH, ND, IB, BV, and MB designed the study, collected the data, analyzed the data, interpreted the data, and wrote the manuscript. IB, LS, and DR processed the samples. MC performed the neuropsychological evaluations. AR performed the statistical analysis. AC, AT, and IB analyzed the data and interpreted the data. All authors revised the manuscript and approved it for submission.

## Conflict of Interest

The authors declare that the research was conducted in the absence of any commercial or financial relationships that could be construed as a potential conflict of interest.

## Publisher's Note

All claims expressed in this article are solely those of the authors and do not necessarily represent those of their affiliated organizations, or those of the publisher, the editors and the reviewers. Any product that may be evaluated in this article, or claim that may be made by its manufacturer, is not guaranteed or endorsed by the publisher.
